# Sage Extract as a Natural Source of Corrosion Inhibitor for Tinplate in 3.0% NaCl

**DOI:** 10.17113/ftb.59.04.21.7026

**Published:** 2021-12

**Authors:** Maja Dent, Regina Fuchs-Godec

**Affiliations:** 1 Faculty of Food Technology and Biotechnology, University of Zagreb, Pierottijeva 6, 10000 Zagreb, Croatia; 2 Faculty of Chemistry and Chemical Engineering, University of Maribor, Smetanova 17, 2000 Maribor, Slovenia

**Keywords:** corrosion, green inhibitor, 3.0% NaCl, sage, *Salvia officinalis*, tinplate

## Abstract

**Research background:**

Due to the growing interest and attention of the world towards environmental problems and protection of environment, the worldwide demand for biodegradable and effective corrosion inhibitors for tinplate has grown. Considering the diversity of the structures of polyphenols that are present in sage extract, it represents a promising potential source of low-cost and effective biodegradable green corrosion inhibitor for tinplate in 3.0% sodium chloride solution which is evaluated in this study.

**Experimental approach:**

Tafel polarisation and electrochemical impedance spectroscopy (EIS) at 25 °C have been used to evaluate the inhibitory activity of sage (*Salvia officinalis* L.) extract as a green inhibitor for the protection of tinplate from corrosion in 3.0% sodium chloride solution.

**Results and conclusions:**

We used electrochemical impedance spectroscopy to show that sage extract could serve as an effective inhibitor (94.1%) of the corrosion of tinplate in 3.0% NaCl at a mass concentration of 0.2 mg/L and temperature of 25 °C. The results obtained from potentiodynamic polarisation reveal that the sage extract acts as a mixed type inhibitor, with inhibition efficiency up to 82.5%, and the inhibition efficiencies calculated from EIS are in close agreement with these results. Attenuated total reflection-Fourier transform infrared spectroscopy (ATR–FTIR) results indicated that the inhibitory effect of the sage extract is due to the presence of a passivation layer on the tinplate surface, which consists of organic compounds such as polyphenols. These results confirm that the sage extract is more efficient in inhibiting the corrosion of tinplate at a concentration of 0.2 mg/L than at higher concentrations. Also, it shows good inhibition of tinplate in 3.0% sodium chloride solution.

**Novelty and scientific contributions:**

The exceptional corrosion inhibition potential of sage extract opens a door for its use and revalorization as a green corrosion inhibitor in the food industry.

## INTRODUCTION

Tinplate cans are used for conserving various meat and fish food products, ready-made meals, pet foods, fats and oils, various fruit and vegetable products, and for packaging of confectionary products, coffee substitute drinks, other powdered food products and food supplements. Nature and addition of sodium chloride to canned food products have highly influenced the behaviour of tinplate. Sodium chloride solution is often used in the packaging of pet food, meat, some vegetable products and fish. Addition of some additives, such as nitrates or nitrites, as well as an increase in temperature can cause corrosion of the tinplate sheet in the sodium chloride solution (*1*). The corrosion occurs as oxygen penetrates the tinplate *via* small pores and fractures on its surface. This causes rusting of steel by formation of a passive oxide layer. The technology applied for the protection of the interior of the can and lid and the type of protective lacquer coating is determined by the content to be packaged in the can because acidic or alkaline effects of some products cause corrosion. Since tinplate is quite important economically for the canning industry and due to its various industrial applications, the protection of tinplate against corrosion has attracted a lot of attention. A small number of studies on the corrosion behaviour of tinplate have been conducted, and the corrosion behaviour in neutral media is still uncertain (*1*-*5*). Therefore, a study of the corrosion behaviour of tinplate in sodium chloride solution is of great significance. Huang *et al*. (*2*) have concluded that the corrosion of tinplate was different from pure tin. Corrosion films on pure tin exposed to sodium chloride solution were composed of SnO_2_, SnO or Sn(OH)_2_. However, no corrosion products have been detected on the tinplate. The alloy layer of tinplate greatly affects the corrosion resistance of electrolytic tinplate. In order to improve the corrosion resistance of tinplate, some studies about the alloy layer of tinplate have been conducted (*1*, *4*). There are several methods to protect tinplate against corrosion and one of them is to add natural inhibitors to the solution in contact with the surface in order to reduce the corrosion rate and inhibit the corrosion reaction. The corrosion protection is achieved by adsorption of inhibitor molecules on the tinplate surface based on two types of adsorption processes, *i.e*. chemical and physical adsorptions. The mechanism of adsorption on the tinplate surface reduces the corrosion rate by increasing or decreasing the cathode or anode reactions and by reducing the diffusion rate of aggressive components on the tinplate surface (*6*). There is a plethora of opportunities for discovering new, economical and eco-friendly corrosion inhibitors from this exceptional source of natural products, such as phytochemicals from plants. The inhibition performance of plant extracts is ascribed to the presence of complex organic species, including phenolic compounds in their composition. Functional groups with conjugated double bonds, nitrogen and oxygen atoms or aromatic rings in their molecular structures, which are the major adsorption centres (*7*), are the integral part of these organic compounds. This is why a new group of natural corrosion inhibitors that have a potential to replace the synthetic ones is being investigated. Several studies on the inhibition of corrosion of iron, steel or carbon steel by means of plant extracts (*7*-*11*) or purified compounds (*12*, *13*) have recently been conducted. Also, research has shown that *Ficus tikoua* leaf extract (*14*) is a good corrosion inhibitor for carbon steel under acidic conditions, while *Rosmarinus officinalis* (*7*), *Ginkgo* (*8*), *Aloe vera* (*10*), *Tinospora crispa* (*11*), sage (*15*), bamboo (*16*), *Thymus vulgaris* (*17*) and *Eucalyptus* (*18*) have been found to act as a very efficient inhibitor of iron or steel corrosion in acidic media. There is a great interest in using natural plant extracts or compounds that can act as cathodic or anodic corrosion inhibitors in neutral or acidic media. However, few studies have been conducted to investigate the corrosion inhibition by natural compounds on tinplate or pure tin used in canning industry. For example, the essential onion oil can be used as an excellent natural inhibitor of tinplate corrosion in canned tomato purée production (*5*). Furthermore, pectin from tomato peel waste acts as a cathodic inhibitor of tin corrosion in 2% NaCl, 1% acetic acid and 0.5% citric acid (*19*). Thus, there are several studies examining the inhibitory properties of natural compounds on tinplate or pure tin. Searching the literature, we have not found any publications on the effect of sage as a natural inhibitor on tinplate corrosion. A research in potentiodynamic polarisation has revealed that *Salvia officinalis* leaf extract (*15*) and sage essential oil (*20*) act as a green corrosion inhibitor of steel in acidic media. Sage (*Salvia officinalis*, Lamiaceae) contains naturally occurring phenolic dimers, flavonoids and plant oils. In this context, considering the diversity of the structures of polyphenols that are present in sage, it represents a promising potential source of so-called green inhibitors.

The purpose of this research is to investigate the inhibitory effect of the sage extract on the corrosion of tinplate in 3.0% sodium chloride solution, by means of potentiodynamic and electrochemical impedance spectroscopy (EIS), attenuated total reflection-Fourier transform infrared spectroscopy (ATR–FTIR) analysis and open circuit potential (OCP). Effects of inhibitor concentration (0.05–0.5 mg/L) and immersion time (1–12 h) were investigated. The aim is to define, as accurately as possible, the anodic and cathodic processes that occur during tinplate corrosion over a specific time frame, and to determine the type of inhibitor in question – whether sage extracts are anodic or cathodic types of inhibitors. The tinplate sample will be observed under the microscope and the results will be used to try and determine if the inhibitory layer of the plant extract on the surface of tinplate is adsorbed in a specific time frame. Likewise, in order to determine the groups of compounds that are adsorbed with time, ATR-FTIR analysis will be used. Reportedly, sage extract still has not been studied for the purpose of corrosion inhibition on tinplate in 3.0% sodium chloride solution. This research will determine the optimal concentration of sage extract for achieving inhibitory activity of tinplate corrosion.

## MATERIALS AND METHODS

### Reagents

Sodium chloride was purchased from Kemika (Zagreb, Croatia). Petroleum ether and ethanol were bought from Carlo Erba (Val de Reuil Cedex, France). The Millipore apparatus was used to obtain deionised water for the preparation of sodium chloride solution.

### Plant material

Naturally growing Dalmatian sage (*Salvia officinalis* L.) is indigenous to the Mediterranean part of Croatia, specifically Pirovac. It was harvested in the evening and dried immediately after harvesting. After that, the dry sage was packed in polyethylene bags and kept in a dark, dry and cool place. Before use, the plant material was crushed using a household blender (Tefal, Mayenne, France) and then used for extraction.

### Tinplate samples

The tinplate used in the experiments was a tin-coated carbon steel with the thickness of the tin coating of about 2.8 g/m provided by the Lim Samoborka Company (Samobor, Croatia). Round tinplate specimens, with the cross-sectional area of 1.14 cm^2^, were placed in a polytetrafluoroethylene (PTFE) holder so that the surface area of 0.875 cm^2^ was exposed to the working solution. The surface was washed with distilled water, degassed by ethanol in an ultrasonic bath and then dried before use in experiments.

### Preparation of plant extract

Dried sage (*Salvia officinalis* L.) leaves were ground to powder. A mass of 4 g of the powder was extracted with 250 mL of petroleum ether in a Soxhlet extractor for 6 h. The extract was concentrated under vacuum at 50 °C until the solvent was completely removed. The remaining extract was stored in a refrigerator for future use. In order to prepare the desired concentrations by dilution with 3.0% sodium chloride solution, stock solutions of sage extract were used. The concentrations of sage extract were 0.05, 0.1, 0.2, 0.3, 0.4 and 0.5 mg/L in a 3.0% sodium chloride solution.

### Electrochemical measurements

The electrochemical measurements of the influence of sage extracts on the corrosion of tinplate in 3.0% sodium chloride solution were carried out using a potentiostat/galvanostat ZRA (Gamry Instruments Inc, Warminster, PA, USA). A platinum electrode as a counter electrode and saturated calomel electrode (SCE) as a reference electrode were used in electrochemical experiments carried out in an electrolytic cell. The tinplate sample embedded in the PTFE holder served as a working electrode. Before each experimental run, the working electrode (tinplate samples) was washed with distilled water and degassed with ethanol in an ultrasonic bath. Then, in order to obtain a stable state, the electrode was immersed in a test solution at open circuit potential (OCP) for 1 h. The potentiodynamic current potential curves were recorded by automatically changing the electrode potential from -0.6 to no more than -0.1 V with a scanning rate of 1 mV/s. EIS measurements were carried out within the 100 kHZ–1 mHz frequency range at a steady OCP disturbed by an amplitude of 10 mV. The working electrode was immersed in the solution for 2 h in order to allow stabilisation of the stationary potential. Nyquist and polarisation plots were then generated from the individual measurements. The Tafel method of extrapolation and Faraday laws were used to determine corrosion parameters *via* ZView software (Scribner Associates, Southern Pines, NC, USA), while impedance parameters were ascertained through appropriate electric circuit models. In order to ensure that reproducible results were reported, each experiment was repeated no less than three times. All electrochemical results were carried out at 25 °C. The kinetic parameters measured during corrosion processes were used to calculate the surface coverage *θ*, polarisation resistance *R*_p_ and the corrosion current density *j*_corr_.

### ATR–FTIR spectroscopic analysis

Attenuated total reflection–Fourier-transform infrared (ATR–FTIR) analysis (IRAffinity-1; Shimadzu, Kyoto, Japan) was used to characterise 3.0% sodium chloride solution with the addition of 0.2 mg/L sage extract and the protective adsorption film modified on the tinplate after its immersion in the solution mentioned above at room temperature for 12 h. The spectral resolution was 4 cm^-1^ and a wave number range of 400–4000 cm^-1^ was used for the collection of IR spectra and identification of protective film formation through a comparison with the standard peak positions of the groups.

### Scanning microscope

The tinplate samples were immersed for 12 h in two different 3.0% sodium chloride solutions; one with and another without the addition of 0.2 mg/L of inhibitor (sage extract). After that, the tinplate sample was removed, rinsed quickly with distilled water and dried. The changes on the tinplate surface were examined under the microscope (model BX51; Olympus, Center Valley, PA, USA).

## RESULTS AND DISCUSSION

### Corrosion resistance of the tinplate exposed to 3.0% NaCl with sage inhibitor

Open circuit potential (OCP) test, the potentiodynamic polarisation method and electrochemical impedance spectroscopy (EIS) techniques were used to investigate the corrosion behaviour of tinplate in a 3.0% sodium chloride solution containing different concentrations (0.05–0.5 mg/L) of sage extract at 25 °C. In order to allow stabilisation of the steady-state potential, electrochemical polarisation was started 60 min after the immersion of the working electrode in the solution in all experiments.

Table 1 shows that OCP values shifted to a more positive potential with time when sage extract was added to the blank (3.0% NaCl) solution from -502.9 to -428.7 mV. The shift of the steady state value of OCP in the presence of sage extracts was about 73.2 mV, which suggests that all sage extracts act as mixed type corrosion inhibitors.

**Table 1:** Kinetic parameters for corrosion of tinplate obtained from potentiodynamic polarisation curves with and without the addition of sage extract to 3.0% NaCl at 25 ºC

*γ*(sage extract)/ (mg/L)	*E*_corr_/mV	*j*_corr_/(μA/cm^2^)	*v*_corr_/(mm/year)	*β*_c_/mV	*β*_a_/mV	*θ*	*η*/%
Blank	-502.9	2.880	0.0338	438.8	55.24	-	-
0.05	-518.6	2.261	0.0265	197.1	54.40	0.215	21.5
0.1	-521.6	1.521	0.0183	255.0	44.99	0.472	47.2
0.2	-428.7	0.503	0.0059	251.8	18.53	0.825	82.5
0.3	-561.7	0.599	0.0070	309.4	-	0.792	79.2
0.4	-454.4	0.869	0.0102	333.1	28.85	0.698	69.8
0.5	-493.6	0.962	0.0113	370.3	51.47	0.666	66.6

*j*_corr_=corrosion current density, *β*_c_ and *β*_a_=anoidic and cathodic Tafel slope, *θ*=surface coverage, *η*=inhibition efficiency

**Fig. 1:**
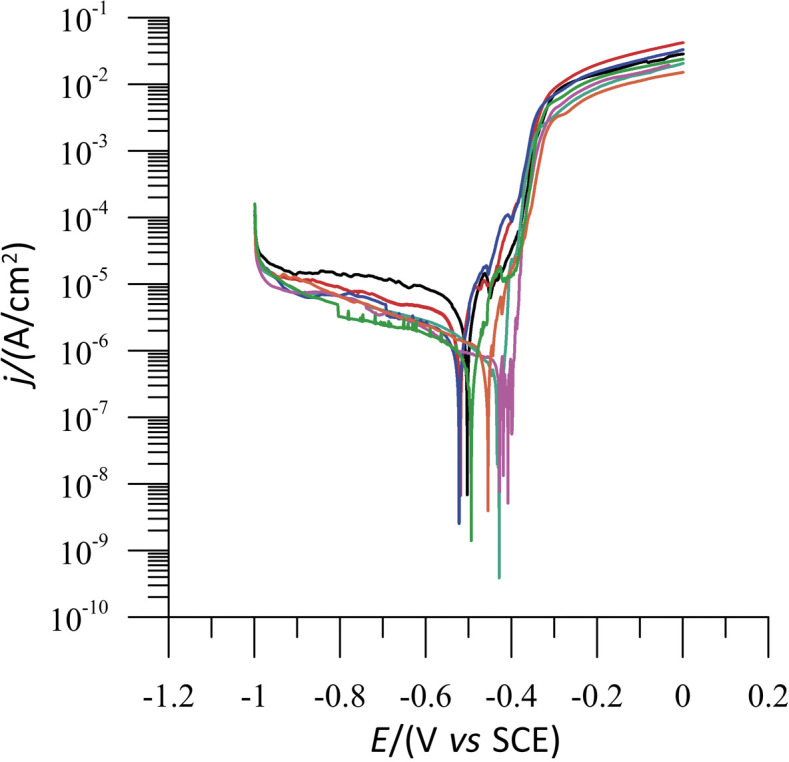
Potentiodynamic polarisation curves (1 mV/s) for tinplate in 3.0% NaCl solution at 25 °C with the addition of *γ*(sage inhibitor)/(mg/L): 0 (black), 0.05 (red), 0.1 (blue), 0.2 (turquoise), 0.3 (violet), 0.4 (orange) and 0.5 (fluorescent green)

As previously established, a difference higher than 85 mV in OCP value is recognized as a classification evidence of a compound as an anodic or a cathodic type of inhibitor (*17*, *21*, *22*). Therefore, the sage extract acts as a mixed type inhibitor since the values of corrosion potential do not change significantly in the cathode or the anode direction (*23*). Fig. 1 shows typical Tafel plot for the corrosion of tinplate in 3.0% NaCl and in the presence of sage extract at different concentrations (0.05–0.5 mg/L), whereas their electrochemical parameters are given in Table 1. There was a decrease of the corrosion current density (*j*_corr_) values in the presence of sage extract up to the concentration of 0.2 mg/L in 3.0% NaCl, which means that the corrosion process of tinplate was suppressed (Table 1). However, with further increase of sage extract concentration, current density slightly increases suggesting that the concentration of 0.2 mg/L has stronger inhibitive properties than other concentrations of the extract. It is evident from Table 1 that the highest inhibition efficiency was obtained at the concentration of 0.2 mg/L sage extract in 3.0% NaCl (82.5%).

Results clearly show that the addition of sage extract to the 3.0% NaCl solution at a concentration of 0.2 mg/L results in a small shift in the corrosion potential to the less negative values, as well a shift in both anodic and cathodic branches towards lower current densities. The decrease of *j*_corr_ of all sage extracts in comparison with 3.0% NaCl may suggest mixed type corrosion inhibition behaviour with a predominant decrease at the cathodic site (*17*, *24*).

The confirmation was also supported by a substantial difference of the cathodic Tafel slope (*β*_c_) in the presence and absence of sage extract in 3.0% NaCl. Furthermore, the shift of *E*_corr_ was 73.2 mV, hence the inhibitor can be seen as a mixed type inhibitor.

In 3.0% NaCl solution, the presence of 0.2 mg/L sage extract causes a notable drop in the corrosion rate, *i.e.* shifts both anodic and cathodic curves to lower current densities, which is evident from Tafel plots. Inhibitor efficiency of sage extract at 0.2 mg/L reaches up to a maximum of 82.5%. The adsorption of the inhibitor (sage extract) on the active sites of tinplate surface blocks the access of aggressive species to the metal surface, decreases the dissolution of the tinplate surface area and retards the corrosion process of tinplate without any changes to the reaction mechanisms.

**Fig. 2:**
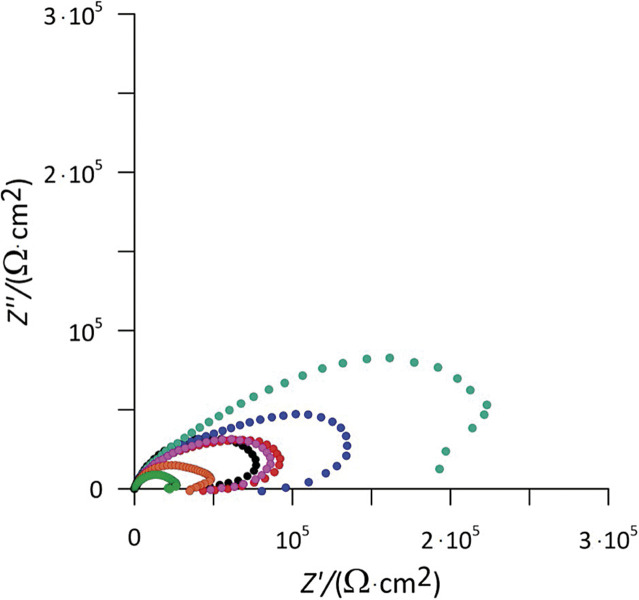
Nyquist plots for tinplate in 3.0% NaCl solution at 25 °C with the addition of *γ*(sage inhibitor)/(mg/L): 0 (black), 0.05 (red), 0.1 (blue), 0.2 (turquoise), 0.3 (violet), 0.4 (orange) and 0.5 (fluorescent green)

Impedance measurements of tinplate at its OCP after one-hour immersion in 3.0% NaCl solution alone and in the presence of different inhibitor concentrations were performed in a frequency range of 100 kHz-1 mHz. Nyquist diagrams of tinplate in 3.0% NaCl are shown in Fig. 2 to compare the corrosion behaviour in the presence of different concentrations of the used inhibitor. The high frequency semicircle is attributed to the time constant of charge transfer and double-layer capacitance, while the presence of a low-frequency inductive loop can be attributed to the relaxation process resulting from the adsorption of inhibitor on the tinplate surface.

**Fig. 3:**
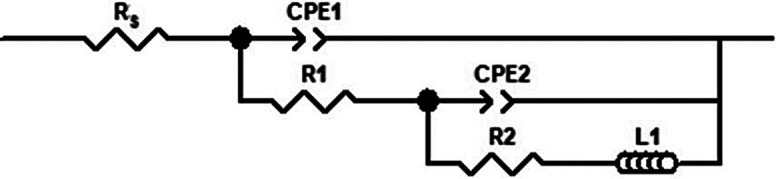
Equivalent circuits for tinplate samples in 3.0% NaCl with and without the addition of sage (*Salvia officinalis* L.) extract. *R*_s_=resistance of solution, *R*_1_=resistance of the adsorbed film and *R*_2_=charge transfer resistance, CPE_1_=constant phase element connected to the capacitance of the adsorbed film, CPE_2_=constant phase element of the double layer connected to the double layer capacitance, L_1_*=*inductive element connected in parallel

To analyse the measured EIS data, a suitable electrical equivalent circuit has to be chosen. The obtained experimental data were fitted using the electrical equivalent circuit (EEC) shown in Fig. 3 and the ZView software (Scribner Associates).

It can be seen from the Nyquist diagrams (Fig. 2) that the depressed semicircles are present in both cases where the electrodes are not inhibited and where a protective adsorption film was obtained on the tinplate surface. Such a phenomenon is usually observed at the practical metal electrode/solution interface, which is known to be related to the roughness of the electrode surface. Metals usually corrode in the NaCl solutions, increasing the roughness of the electrode surface and the use of a constant phase element (CPE) is required instead of the ideal capacitor in the EEC.

In the used EEC, *R*_s_ represents the resistance of the solution. Furthermore, the equivalent circuit consists of two RC (circuit containing resistance and capacitance) circuit elements, where *R*_1_ is the resistance of the adsorbed film and CPE_1_ is the constant phase element connected to the capacitance of the adsorbed film. CPE_2_ is the constant phase element of the double layer connected to the double layer capacitance *C*_dl_ in parallel with the charge transfer resistance (*R*_2_), which is in series with the inductive element L_1_ connected in parallel. The electrochemical parameters obtained from the EIS measurements after 2.5 h of immersion, including the percentage of inhibition efficiency, are shown in Table 2.

**Table 2:** Equivalent circuit parameters for tinplate electrode in corrosive 3.0% NaCl solution in the presence and absence of inhibitors after 2.5 h of immersion at 25 °C

***γ*(sage extract)/** **(mg/mL)**	***R*_1_/** **(kΩ∙cm^2^)**	**n_1_**	***C*_1_/** **(μF/cm^2^)**	***R*_2_/** **(kΩ∙cm^2^)**	**n_2_**	***C*_2_/** **(μF/cm^2^)**	***R*_p_/** **(kΩ∙cm^2^)**	***η*/%**
Blank	8.17	0.815	4.44	19.91	0.646	2.29	19.91	
0.05	19.02	0.825	68.26	98.24	0.503	2.82	117.26	83.02
0.1	45.76	0.830	88.50	101.50	0.538	3.61	147.26	86.48
0.2	40.76	0.752	101.83	298.70	0.559	2.84	339.46	94.13
0.3	31.47	0.753	117.63	80.39	0.524	6.56	111.85	82.20
0.4	9.18	0.789	6.84	87.82	0.776	5.02	90.00	77.88
**0.5**	**9.05**	**0.825**	**6.90**	**49.97**	**0.651**	**3.12**	**59.02**	**66.27**

*R*_1_=charge transfer process, *R*_2_=impedance with the interface reaction between the film and the substrate, *R*_p_=polarisation resistance (*R*_1_+*R*_2_), n_1_ and n_2_=quantify various physical phenomena of the surface such as roughness, adsorption of inhibitor molecules, formation of the porous layer, *C*_1_ and *C*_2_=capacitance, *η*=inhibition efficiency

Keddam *et al.* (*25*) have reported that the inductive loops in the low-frequency range are generally attributed to the existence of relaxation processes of adsorbed species (*e.g.* relaxation of coverage by an adsorbed intermediate). They have also been associated with the modulation of surface or salt film properties. In a paper on exfoliation corrosion of aluminium alloys, induction loops have been associated with the weakening of the protective effect of the aluminium oxide layer due to anodic dissolution of the alloy.

The presence of L_1_ in the impedance spectra when the inhibitor is added suggests that the tinplate could be dissolved (to a lesser extent, indicating relatively high values of polarisation resistance) by a direct charge transfer at the surface with adsorbed sage extract.

As it can be seen from Table 3, the polarisation resistance *R*_p_ (which is the sum of all resistances contained in the EEC) values increased with the increase of inhibitor concentration from 0.05 to 0.2 mg/L. The biggest semicircle diameter was obtained with the addition of 0.2 mg/L of sage inhibitor, with a value close to 298.70 kΩ·cm^2^ after 2.5 h of testing, several times bigger than the one obtained in the uninhibited solution (*17*).

**Table 3:** Polarisation resistance (EIS measurements) and inhibition efficiency for tinplate in 3.0% NaCl with and without inhibitor addition after different immersion time

*γ*(sage extract)/(mg/L)	*t*(immersion)/h	*R*_p_/(kΩ∙cm^2^)	*η*/%
	2.5	19.91	-
	5	11.60	-
Blank	8	7.40	-
	10	5.94	-
	12	4.76	-
	2.5	98.24	83.0
	5	24.30	52.3
0.05	8	11.52	35.8
	10	9.01	34.0
	12	9.01	47.1
	2.5	101.50	86.5
	5	60.49	80.8
0.1	8	25.76	71.6
	10	13.61	56.4
	12	8.46	43.7
	2.5	298.70	94.1
	5	133.20	91.3
0.2	8	67.07	89.0
	10	41.70	85.8
	12	25.82	81.6
	2.5	80.39	82.2
	5	27.45	57.7
0.3	8	16.78	55.9
	10	13.81	57.0
	12	13.71	65.3
	2.5	87.82	77.9
	5	25.87	55.2
0.4	8	16.49	55.1
	10	13.57	56.2
	12	12.13	60.7
	2.5	49.97	78.0
	5	72.61	84.0
0.5	8	46.73	84.2
	10	33.15	82.1
	12	28.67	66.3

*R*_p_=polarisation resistance, *η*=inhibition efficiency

The reason for this might be any kind of increase in the surface coverage by the inhibitor, which led to an increase in the inhibition efficiency. It can also be argued that an increase in semicircle size with the increase of inhibitor concentration (as shown in Fig. 2) also means a reduction in corrosion rate, which agrees well with the results of potentiodynamic measurements.

A further increase in the concentration of an added inhibitor leads to a marked decrease in the radius of depressed semicircles in the Nyquist plots, which is also reflected in a decrease in inhibitory efficiency. In order to explain this effect, it could be speculated that an excessive concentration of inhibitor may impede the movement of inhibitor molecules (*7*, *26*), possibly even leading to the formation of larger structures or complexes with dissolved metal ions, which could make transport to the metal surface more difficult.

Another possible explanation would be that above a certain concentration of the inhibitor, there are no more active sites available for adsorption. Therefore, the inhibitor molecules cannot adsorb to the metal substrate. However, corrosion still occurs (which means the inhibitory layer did not change, but corrosion activity increased with immersion time). This is why the efficiency decreased.

It has been previously reported that capacitance values corresponding to the adsorption (or desorption) process are of around 100-1000 times higher than *C*_dl_ (*27*). A significant increase in capacitance was observed at inhibitor concentration between 0.05–0.3 mg/L, ranging from 68.28 to 117.63 μF/cm. In this concentration range the radius of the depressed semicircles also increased sharply compared to the uninhibited surface. Thus, we confirmed the presence of an adsorbed protective film, which is also accompanied by a significant increase in the polarisation resistance and, consequently, a rise in inhibition efficiency.

However, at the inhibitor concentration >0.03 mg/L, the capacitance of the adsorbed film again decreased sharply, indicating a problem with adsorption and thus lower inhibition efficiency values. It should be noted that this study has once again demonstrated the importance of optimizing the concentration of the added inhibitor to avoid activation rather than inhibition.

Table 3 also shows that as the exposure time of the tinplate samples to the corrosive medium with and without the presence of the inhibitor increases, the inhibition effect starts to decrease. Obviously, the dynamics of the anodic process could have a destructive effect on the self-assembling adsorption layer, reducing the possibility of a more successful adsorption of inhibitor molecules.

**Fig. 4:**
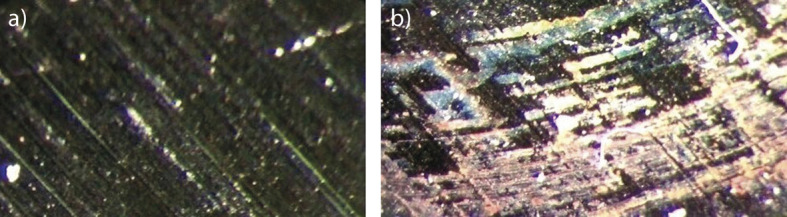
Micrographs of tinplate surface: a) before immersion, and b) after 12 h of immersion at 25 °C in 3.0% NaCl with 0.2 mg/L sage inhibitor

**Fig. 5:**
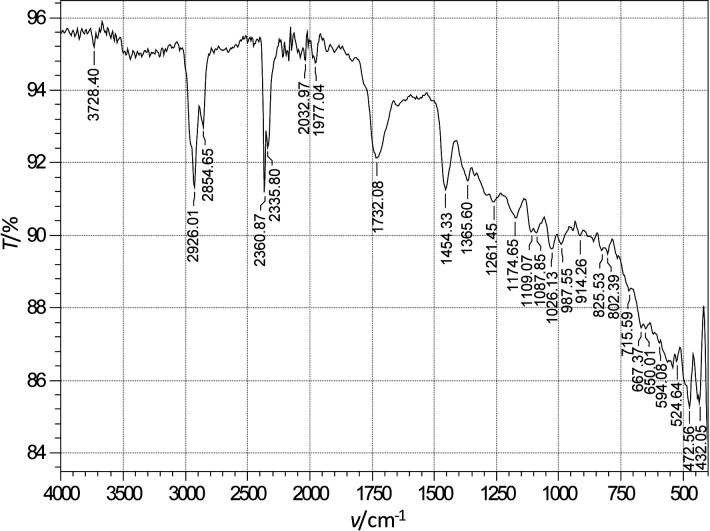
FTIR spectra of tinplate surface after 12 h of immersion at 25 °C in 3.0% NaCl with 0.2 mg/L sage inhibitor

It is evident from the results that after the addition of sage extract to 3.0% NaCl, the impedance response of tinplate has changed. The surface coverage by sage inhibitor of the tinplate after its immersion in 3.0% NaCl containing sage inhibitor is shown in Fig. 4. It has been confirmed by these results that the sage extract at a concentration of 0.2 mg/L has good inhibition properties. ATR–FTIR spectra were used to confirm the protective adsorption film formed on the tinplate surface after exposure to a 3.0% sodium chloride solution containing 0.2 mg/L sage extract (Fig. 5). **FIG. 4****, ****FIG. 5**

### Optical microscopic studies

Optical images of tinplate morphology before immersion and after the corrosion test in 3.0% sodium chloride solution with the addition of the sage extract in a concentration of 0.2 mg/L are shown in Fig. 4. A layer of inhibitor is clearly visible on the tinplate surface due to the presence of the sage extract. The changes in the morphology of tinplate surface in Fig. 4 are the result of the electrochemical action of sage extract inhibition on tinplate surface. The sage extract formed a protective adsorption film on the tinplate surface, inhibiting the diffusion of corrosive anions in the sodium chloride solution.

### Characterization of sage extract

ATR–FTIR analysis was utilised to confirm the presence of some functional groups present in the prepared protective adsorption film on the tinplate surface which act as active corrosion inhibitors. Fig. 5 shows the ATR–FTIR spectra of the protective adsorption film modified on the tinplate surface after exposure to the 3.0% sodium chloride solution containing 0.2 mg/L sage extract. The peak at 3728.40 cm^-1^ is attributed to N–H or O–H stretching. The bands at 2926.01 and 2854.65 cm^-1^ are related to C–H stretching vibration. The peak at 1732.08 cm^-1^ is assigned to C=C or C=O stretching vibration bands, which correspond to stretching modes of carbonyl groups, and one more vibration at 1026.13 cm^-1^ due to a single bond (C–O). The C–H bending bands in  –CH_2_ and CH_3_ are found to be at 1454.33 and 1365.60 cm^-1^, respectively. Besides these, there are adsorption bands at 1261.45, 1174.64, 1109.07, 1087.75 and 1026.13 cm^-1^ attributed to stretching modes of C–O stretching vibrations. The absorption bands below 1000 cm^-1^ can be assigned to the stretching mode of aliphatic or aromatic C–H groups. The presence of O–H, C–H and C=O bonds is evident, which suggests the presence of carboxylic acids. The phenolic components possess heteroatoms, such as N and O, that can act as adsorption centres. All these characteristics correspond to compounds such as phenolic and fatty acids, mono- and diterpenes and flavonoids. These results indicate that the sage extract adsorbed on tinplate surface contains O and N atoms in functional groups and aromatic rings, which is in accordance with other authors (*3*, *7*, *16*, *17*). Fang *et al.* (*12*) conducted a research in which the aromatic structure of phenolic compounds was attributed to corrosion inhibition properties. This FTIR spectrum shows that the sage extract contains a mixture of compounds, *i.e.* flavonoids, fatty acids and mono- and diterpenes. It can be seen from the results of FTIR characterization that the reason for anticorrosion property of sage extract is the presence of O atoms in flavonoid aromatic rings; the band shifting confirms that the sage extract inhibits corrosion by adsorption to the tinplate surface. Sage extract has not yet been used as a corrosion inhibitor for tinplate, but this research has shown evidence of good inhibition properties of sage against tinplate corrosion, due to its chemical composition and high amount of phenolic compounds. The efficiency of sage extract in inhibiting the corrosion of tinplate is attributed to the synergistic effect of several organic molecules present in the extract.

## CONCLUSIONS

Sage extract has not yet been used as a corrosion inhibitor for tinplate, but this research has shown compelling evidence of good inhibition properties of sage against tinplate corrosion, due to its chemical composition. The sage extract inhibits the tinplate in a corrosive medium, in this case 3.0% sodium chloride solution, and the corrosion rate decreases with the increase in sage concentration to 0.2 mg/L. Electrochemical impedance spectroscopy analysis revealed that by increasing the concentration of sage extract in 3.0% NaCl to 0.2 mg/L, the inhibition efficiency increased to 94.1% after 2.5 h. The Tafel polarisation and inhibition efficiency of 82.5% were in reasonably good agreement, while the sage extract showed a mixed type inhibition effect. The inhibitory activity of the sage extract was attributed to the adsorption of organic compounds, such as polyphenolic compounds, to the tinplate surface, thereby blocking the active corrosion sites. Our results show that sage extract in sodium chloride solution can find its application in food packaging industry as an effective mixed type corrosion inhibitor for tinplate that would replace the use of synthetic ones.
